# Japanese Alcoholic Beverage and All Cause Mortality in Japanese Adult Men

**DOI:** 10.2188/jea.11.219

**Published:** 2007-11-30

**Authors:** Motonobu Miyazaki, Hiroshi Une

**Affiliations:** 1 Department of Hygiene and Preventive Medicine, School of Medicine, Fukuoka University.

**Keywords:** alcohol consumption, Japanese sake, all cause mortality, preventive effect

## Abstract

This article examines whether an association is found between quantity of alcohol consumed and all cause mortality, and a relation is detected between types of alcoholic beverage and all cause mortality in Japanese male adults. A cohort study was performed in three towns located in two former coal mine areas and a rural area in Fukuoka. A mail survey was carried out between 1988 and 1990, and was extended in a follow-up period to 1995 (two towns) and 1999 (one town). 6,652 Japanese men aged from 40 to 69 years responded to a questionnaire that included alcohol consumption and smoking habit. The data were analysed with Cox’s proportional hazards model. As regards an association between all cause mortality and quantity of alcohol consumed, statistically significant relations were recognized in occasional drinkers and drinkers with less than 25g ethanol per day (hazard ratio= 0.71, 95% CI: 0.50-0.99; hazard ratio= 0.51, 95% CI: 0.29-0.88, respectively). With respect to drinkers with 25-50g/day and ≧50g/day, no significant relation was observed in the risk for all cause mortality. Comparing all cause mortality for daily drinkers and nondrinkers with respect to the beverage types, a risk was lower for daily drinkers than for nondrinkers with every type of beverage studied. In particular, there was a statistically significant adverse association for all cause mortality among subjects who reported drinking Japanese sake compared with nondrinkers (hazard ratio= 0.45, 95% CI: 0.30-0.68). Alcohol consumption (particularly Japanese sake) is likely to be associated with a reduced hazardous ratio of all cause mortality.

## INTRODUCTION

Many substances are simply bad for health. Smoking is a fitting example. With respect to alcohol consumption, the situation is more complex. The relationship between alcohol consumption and a risk of mortality has been examined about various causes of death. Harmful consequences of alcohol include malignancies, coronary heart disease, stroke, and liver cirrhosis^1^^-^^9^^)^. Epidemiological studies have been conducted to detect a risk of alcohol consumption for health among Japanese^7^^-^^12^^)^. While alcohol consumption is known to bring about many adverse effects, some patterns of intake have been observed to improve health. It is well known that moderate alcohol consumption prevent coronary heart disease^13^^-^^20^^)^. The beneficial effects of alcohol do not seem to be limited to coronary heart disease. Moderate alcohol consumption may also be effective for preventing eye disease^21^^)^ and infectious disease^22^^)^. To reach a balanced view on the effects of alcohol on health, both positive and negative effects on health-related outcomes have to be considered together.

The present study focuses on the risk of alcohol consumption on total mortality. It is well known that the intake of large quantities of alcohol is hazardous to the health and increases mortality from all causes^1^^, ^^2^^, ^^16^^)^. Importance of lifestyle, behavioral, and drinking patterns associated with alcoholic beverage types has been emphasized in examining the differential effects^14^^)^. Little evidence exists, however, about the health effects of drinking patterns beyond the effects of overall consumption^23^^)^, partly because unequivocal global measures are difficult to find^24^^)^. The consumption of different alcoholic beverages is likely to be accompanied not only by different nutritional intake but also other differences in drinker traits and habits. Characteristics correlated with beverage choice may explain the different risks associated with types of beverage in different populations. In this study we examined whether an association is found between quantity of alcohol consumed and all cause mortality, and whether a relation is detected between types of alcoholic beverage and all cause mortality in Japanese male adults.

## SUBJECTS AND METHODS

### Study population

For this cohort study, three towns were selected in two former coal mine areas and a rural area in Fukuoka, the western part of Japan. All coal mines in these areas had been shut down by 1973. Subjects in this study were all males. The total number of the male population aged from 40 to 69 years in these towns was 11,178. These subjects were identified from the municipal population registers. A mail survey was undertaken during 1988 to 1990 with a postal questionnaire, including smoking habit and alcohol consumption. Data on smoking habits included age started, number of cigarettes smoked/day, duration, and age stopped, if pertinent. A self-administered questionnaire was mailed to all male subjects. Participation was voluntary, and informed consent was obtained in each subject.

The follow up period extended from the time of the mail survey to February 1995 (1,902 males in two towns) and to August 1999 (1,750 males in one town). Two hundred and eighty emigrants were identified through the municipal population registers. We identified all causes of death from death certificates for the two areas and the municipal population registers for the one area. Person-years were calculated from the specific date of the mail survey during 1988-1990 until death, emigration, or the end of follow-up (February 28th, 1995 for two towns and August 31st, 1999 for one town). The periods observed were 24,645 person-years for subjects in two towns and 15,796 person-years for subjects in one town.

The smoking habit was grouped into four categories: nonsmokers, ex-smokers, current smokers of 1-19 cigarettes per day (<20 cigarettes/day), and current smokers of 20 cigarettes or more per day (≧20 cigarettes/day).

### Alcohol consumption

A history of alcohol consumption was obtained through the questionnaire. Drinking habit was examined in categories of alcoholic beverage and quantity of alcohol consumed per day. Types of beverage included Japanese sake, beer, and shochu. Usual frequency of intake and usual dose (in unites or bottles) were recorded for each type of beverage. Concerning the categories of alcohol beverage, Japanese sake and beer were classified in a light liquor group; shochu was in a hard liquor group^10^^)^. Subjects were asked whether they were nondrinkers, ex-drinkers, or current drinkers. Ex-drinkers or current drinkers were asked to choose the type of beverage that they usually consume among Japanese sake, beer, shochu or others. They are not allowed to choose more than 1.

We defined nondrinkers as ones who stated that they had never or almost never drunk alcohol in the past. Ex-drinkers were those who had not drunk for a consecutive year at the time of the survey and who indicated previous alcohol drinking. Subjects reported the frequency and the quantity of alcoholic beverage consumption, which was used to clarify them by the following drinking categories; nondrinkers, ex-drinkers (abstainers), occasional drinkers (1 to 3 days per week), and daily drinkers (daily or almost daily). We calculated the unit measurement of alcohol for average daily intake of each beverage on the basis of known alcohol concentration. The average total and daily alcohol consumption were calculated assuming that 180 ml (one unit) of Japanese sake, 630 ml (one bottle) of beer, or 90 ml (one unit) of shochu contain 25g ethanol (which are typical ethanol contents of the Japanese sake, beer, and shochu consumed in Japan). Daily drinkers were classified into three categories; less than 25g per day (<25g/day), 25 to less than 50g per day (25-50g/day), and 50g or more per day (≧50g/day).

### Statistical analysis

We estimated the relationship of smoking and alcohol consumption for all cause mortality by Cox’s proportional hazards model. Nondrinkers were used as the reference group for analyses. Results were expressed as hazard ratios for all cause mortality. In these analyses, age, smoking habit, and alcohol consumption were included as independent variables. All data were analysed with the Statistical Analysis System (SAS) software package (SAS Institute, Cary, NC, USA). “Significant” indicates that 95% confidence intervals (CI) do not include 1.00.

## RESULTS

Table 1 shows the number of subjects and deaths by age groups. Proportions of subjects were almost the same across age groups. Of 11,178 males, 6,785 responded the questionnaire and the response rate was 60.7%. 133 subjects were excluded because of a lack of information on alcohol consumption. Therefore, the participation rate of this study was 59.5% (6,652 subjects). During the study period, 379 deaths were confirmed in the study population.

**Table 1.** Number of subjects and death cases by age.

Age	Number of Subjects	Number of death cases	Person-years
40-49	2,217 (33.3%)	14	14,197
50-59	2,221 (33.4%)	118	13,446
60-69	2,214 (33.3%)	217	12,798

Total	6,652	379	40,441

Notes : Age at baseline.

133 subjects with a lack of information on alcohol consumption were excluded.

Table 2 shows the hazard ratios and their 95% CIs for the risk of all cause mortality by alcohol consumption and smoking habit. In these analyses, age, alcohol consumption, and smoking habit were included as independent variables. As had been expected, age showed an independent relation to a risk for death.

**Table 2.** Hazard ratios and their 95% confidence intervals in study subjects by alcohol consumption and smoking habits for all cause mortality.

Factors	Number of subjects	Number of deaths	Hazard ratios	95% CIs
Age (per 10 years)			2.05	1.77-2.36*

Alcohol intake				
Nondrinkers	1,069	70	1.00	
Ex-drinkers	542	78	2.09	1.51-2.88**
Drinkers				
Occasional	1,475	60	0.71	0.50-0.99*
<25g/day	449	15	0.51	0.29-0.88*
25-50g/day	1,670	78	0.72	0.52-1.00
50g/day≦	1,447	78	0.86	0.62-1.19

Smoking habits				
Nonsmokers	1,463	56	1.00	
Ex-smokers	1,488	84	1.22	0.86-1.71
Current smokers				
<20 cigarettes/day	1,134	87	1.54	1.09-2.16*
≦20 cigarettes/day	2,567	152	1.51	1.11-2.06**

Notes ; Adjusted for other factors in this table

* p < 0.05

** p < 0.01

Of 6,652 subjects, there were 1,069 nondrinkers, 542 ex-drinkers, 1,475 subjects reported drinking occasionally, and 3,566 reported drinking alcohol daily. A similar inverse relation was found between drinkers and their alcohol consumed. Statistically significant adverse relations were recognized in occasional drinkers and drinkers with <25g/day (hazard ratio= 0.71, 95% CI: 0.50-0.99; hazard ratio= 0.51, 95% CI: 0.29-0.88, respectively). The hazard ratio was 2.09 for ex-drinkers compared with nondrinkers (95% CI: 1.51-2.88), although we did not obtain data on quantity of alcohol consumed when ex-drinkers had been drinkers. There was a marginal significant relation between drinkers with 25-50g/day and a risk of all cause mortality. No significant relation was, however, observed between drinkers with ≧50g/day and a risk for all cause mortality. These statistical associations were not changed before and after adjustment for body mass index (kg/m^2^) (data not shown).

The smoking-mortality relation was present. After the adjustment for age and alcohol consumption, hazard ratios for all cause mortality about current smokers with <20 cigarettes per day and with ≧20 cigarettes per day were 1.54 (95% CI: 1.09-2.16) and 1.51 (95% CI: 1.11-2.06), respectively.

Table 3 shows the hazard ratios and 95% CIs for the risk of all cause mortality by types of beverage in daily drinkers. 171 subjects usually drunk wine or whisky were excluded from an analysis because of a small number. Therefore, a total of 3,395 subjects reported drinking alcohol daily and they had a clear preference for only one of beverage types; of these preferrers, 1,123 (33.1%) usually chose Japanese sake, 1,606 (47.3%) usually chose shochu, and 666 (19.6%) usually chose beer. Daily intake was inversely related to the risk for all cause mortality. In particular, there was a statistically significant adverse association for all cause mortality among subjects who reported drinking Japanese sake compared with nondrinkers (hazard ratio= 0.45, 95% CI: 0.30-0.68). In contrast, no statistically significant effect was found in beer preferrers and shochu preferrers.

**Table 3.** Hazard ratios and their 95% confidence intervals for all cause mortality in daily drinkers by types of alcoholic beverage.

Factors	Number of subjects	Number of deaths	Hazard ratios	95% CIs
Nondrinkers	1,069	70	1.00	
Daily drinkers				
Japanese sake	1,123	33	0.45	0.30-0.68***
Beer	666	20	0.66	0.40-1.09
Shochu	1,606	110	0.96	0.71-1.30

Notes ; Adjusted for age, smoking habits and quantity of alcohol consumed.

171 subjects usually drinking wine or whisky were excluded.

*** p < 0.001

## DISCUSSION

Several repots show the protective effects of alcohol consumption: Moderate alcohol consumption shows protective effects from coronary heart disease^13^^-^^20^^)^, stroke^13^^, ^^20^^, ^^26^^)^, cancer mortality^12^^)^, and Helicobacter pylori infection^22^^)^. These epidemiological reports investigated the risk and benefit of alcohol consumption for diseases by types of beverage and/or quantity of alcohol consumed.

At the onset, we hypothesized that alcohol consumption was a relevant factor for all cause mortality. Many population based studies have shown J-shaped or U-shaped association between the amount of alcohol intake and all cause mortality^1^^, ^^2^^, ^^12^^, ^^16^^)^. It is unclear, however, whether drinking pattern have specific health consequences beyond total alcohol consumption. Japanese sake, shochu and beer are the most common alcoholic beverages in Japan^11^^)^. We are especially interested in Japanese sake intake as common alcohol in Japan. Wine, for example, still holds a fairly narrow share of the market^11^^)^.

Most studies, which have been performed in men, concluded that the most apparent beneficial effects of light to moderate drinking are found in men over the age of 40 years^16^^)^. In this study the risks for all cause mortality were lower among current drinkers, and especially statistically significant differences were observed in occasional drinkers (p<0.05) and daily drinkers with <25g/day (p<0.05), whereas a hazard ratio for ex-drinkers increased significantly (p<0.01). The risk of daily drinkers with ≧50g/day was lower than the risk for nondrinkers. This may be because some drinkers, out of drinkers with ≧50g/day, quit drinking for some health problems and classified into ex-drinkers. Several reports show a relationship between consumption and all cause mortality: the higher the consumption, the higher the risk of harmful outcome^1^^, ^^2^^, ^^12^^, ^^15^^, ^^26^^)^. Therefore, a study should be focused in the next extension of follow up time for daily drinkers to distinguish the effect of alcohol intake.

A more interesting finding was the association between all cause mortality and the types of beverage. A few studies concluded that beer-drinking men were at the low risk of hospitalization than others^27^^)^ and were at the low risk for coronary heart disease^14^^, ^^25^^)^. Several studies showed that alcohol itself, rather than a type of beverage, is responsible for the reduction in the risk^14^^, ^^16^^)^. Although it was not statistically significant, men who daily consumed beer or shochu appeared to be less likely to be at a high risk for all cause mortality compared with nondrinkers. Daily Japanese sake intake was statistically adversely associated with a risk of all cause mortality (p<0.001). Our findings suggest that the benefit of daily alcohol consumption seems to help maintaining at a low risk for all cause mortality.

In this study we focused a relationship between all cause mortality and types of beverage. Light alcohol consumption is likely to be associated with reduced hazardous ratios of all cause mortality. Our data indicate that intake of Japanese sake is associated with a low risk of all cause mortality. Since Japanese sake is the special Japanese beverage, its consumption often accompanies simultaneous intake of Japanese typical foods^27^^)^. It is reported that Japanese sake may be protective effect against mutagens^28^^)^. However, we could not determine whether these results were due to component in Japanese sake in addition to ethanol or whether this was an effect due to intake of ethanol with meals. There are important areas for future research.

Although our study was conducted in the three towns, there were little differences between each town about results analysed on all cause mortality and alcohol consumption (data not shown). However, a limitation of our study was the fact that information on alcohol consumption was obtained by a self-administered questionnaire without verifying by biological markers. It was possible that the true alcohol consumption was underreported, especially among heavy drinkers^29^^)^. However, we believe that alcohol consumption has probably been underreported to some extent, because the true higher risk in the group with high alcohol consumption related to actually higher alcohol consumption. For a result of the Japanese sake, underreporting would consequently reflect that daily consumption of Japanese sake is likely to be beneficial.

Although we could not clarify why risks of occasional and daily drinkers with <25g/day were low, we arrived at the following conclusion in our study: The risks for all cause mortality were lower among occasional drinkers and daily drinkers with <25g/day. A protective effect of alcohol consumption was likely to be in Japanese sake preferrers, but the effect of alcohol consumption was significant only in controlling for age, sex, and smoking. Therefore, an observation period in our study needs to be extended in the future to investigate associations between a type of beverage and its quantity of intake about each alcoholic beverage. Such findings will have impact on the alcoholic beverage industry, the public health policy makers, and the consumers at large. Further study is needed to address observed associations in Japanese male adults and should aim to obtain a more complete understanding of their underlying mechanisms.
